# Barriers and Facilitators to Integrating Health Service Responses to Intimate Partner Violence in Low‐ and Middle‐Income Countries: A Comparative Health Systems and Service Analysis

**DOI:** 10.1111/sifp.12021

**Published:** 2017-04-19

**Authors:** Manuela Colombini, Colleen Dockerty, Susannah H. Mayhew

## Abstract

This systematic review synthesizes 11 studies of health‐sector responses to intimate partner violence (IPV) in low‐ and middle‐income countries. The services that were most comprehensive and integrated in their responsiveness to IPV were primarily in primary health and antenatal care settings. Findings suggest that the following facilitators are important: availability of clear guidelines, policies, or protocols; management support; intersectoral coordination with clear, accessible on‐site and off‐site referral options; adequate and trained staff with accepting and empathetic attitudes toward survivors of IPV; initial and ongoing training for health workers; and a supportive and supervised environment in which to enact new IPV protocols. A key characteristic of the most integrated responses was the connection or “linkages” between different individual factors. Irrespective of their service entry point, what emerged as crucial was a connected systems‐level response, with all elements implemented in a coordinated manner.

Intimate partner violence (IPV) is a recognized public health concern (Devries et al. 2013; WHO, LSHTM, and SAMRC 2013). IPV is defined as the reported experience of one or more acts of physical and/or sexual violence by a current or former sexual or marital partner (WHO, LSHTM, and SAMRC 2013). Most definitions include psychological violence and coercive control (e.g., humiliation, isolation, asset control, threats, aggression, stalking) by current or former partners (Saltzman et al. 1999; Dutton et al. 2006; Campbell et al. 2008). Recent global estimates suggest that 30 percent of women experience IPV during their lifetime, with higher estimates of 37 percent in sub‐Saharan Africa, Southeast Asia, and Eastern Mediterranean regions. The next highest prevalence is reported in the Americas (Canada, Latin America, and the United States), with approximately 30 percent of women reporting lifetime exposure (WHO, LSHTM, and SAMRC 2013). A range of adverse physical and mental health outcomes are also associated with IPV, including injuries, adverse birth outcomes, substance misuse, suicide, and HIV infection (Abramsky et al. 2011; WHO, LSHTM, and SAMRC 2013).

For the health sector in low‐ and middle‐income countries (LMICs) to meet the multiple complex needs of women experiencing IPV, a coordinated, comprehensive, and well‐integrated response is needed (García‐Moreno et al. 2015). Few studies exist that analyze the factors that enable or constrain comprehensive and integrated IPV responses. Furthermore, while there is considerable literature examining the effectiveness of potential health interventions for IPV (Colombini, Mayhew, and Watts 2008; Feder et al. 2011; O'Campo et al. 2011; Taft et al. 2013; Bair‐Merritt et al. 2014; O'Doherty et al. 2014), these failed to explore and systematically document the wider health‐systems factors and processes that could affect the integration of such interventions into routine health care services. We therefore have conducted a systematic review (of quantitative and qualitative studies) to identify and analyze barriers and facilitators to integrated health sector responses to intimate partner violence in LMICs. In particular, our study explores interventions by service entry point and maps the different benefits and challenges derived from each particular entry point.

## METHODS

We conducted a systematic literature review of health services responses to IPV, following the Preferred Reporting Items for Systematic Reviews and Meta‐Analyses (PRISMA) guidelines (Moher et al. 2009).

### Search Strategy

We conducted a literature search to generate evidence on health sector responses to IPV in LMICs and the integration of those responses. The following databases were searched: Medline, Embase, Cumulative Index to Nursing and Allied Health Literature (CINAHL), Web of Knowledge, and Global Health. Because of both the large volume of articles retrieved and time constraints, Popline, WHO Library Information System, and DFID (Research for Development) databases were not searched. The search terms were aimed at retrieving articles that discussed four concepts: IPV, health sector, health sector responses to address IPV, and LMIC. The full list of search terms and strategies for each database can be found in Appendix 1.^1^ Reference lists of selected articles were hand‐searched to extract additional relevant articles. Reference lists of key grey literature reports were also searched, and relevant articles were extracted (Colombini, Mayhew, and Watts 2008; Bott et al. 2010; Ward 2011; WHO 2013).

#### Inclusion and Exclusion Criteria

We designed and revised inclusion and exclusion criteria. Criteria were developed to retrieve articles that discussed the processes of implementation of a health sector response to IPV and the integration of multiple aspects of a health sector response to IPV. All literature identified by the search strategy was screened for relevance using the inclusion and exclusion criteria shown in Table 1.

**Table 1:** Inclusion and exclusion criteria

	Inclusion criteria	Exclusion criteria
Topic	A health sector intervention for IPV; responses include any one or a combination of the following elements: screening, identification, treatment, documentation, support, or referral for women who have experienced IPV, health worker training, protocols, development, and implementation of guidelines or policies addressing IPV	Prevalence of GBV or IPV Female genital mutilation, trafficking, transactional sex IPV as a cause, risk factor, exposure, association, or outcome Risk factors or protective factors for GBV Perceptions of and attitudes toward GBV Health care experiences of survivors of violence Knowledge, attitudes, and practices of health workers Validation, reliability, acceptability, or feasibility test of an IPV screening tool Non‐health‐sector interventions addressing GBV/IPV Interventions addressing only sexual violence
Participants	Adult women	Children, men, elderly, lesbian, gay, bisexual, transsexual, or queer (LGBTQ), sex workers, people with disabilities
Setting	IPV in nonconflict, humanitarian, or emergency setting LMIC as defined by World Bank (2014) Health sector setting	GBV in conflict, humanitarian, or emergency regions High‐income country Pre‐qualification training Any interventions/studies outside health settings
Study design	Peer‐reviewed qualitative, quantitative, or case studies	Editorial and commentary, single‐case studies, thesis or dissertation; non‐peer reviewed
Timeline	After 2000	Before 2000
Language	English	Non‐English

#### Screening and Quality Appraisal

**Figure 1:**
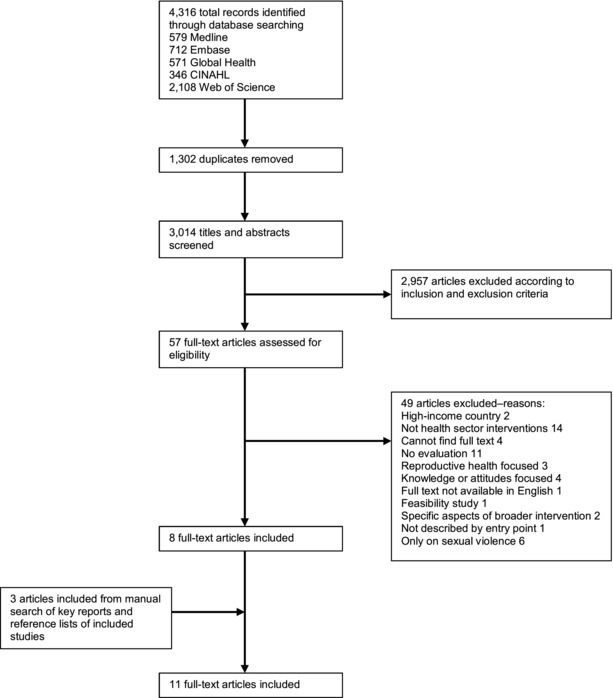
**Literature search results**

After searching all five databases with the search terms, 4,316 articles were retrieved from first record to 10 November 2015. Retrieved articles were exported to Endnote and duplicates were removed, leaving 3,014 articles. Titles and abstracts were screened to search for articles relevant to the research question based on inclusion and exclusion criteria. Full texts of the remaining 57 articles were screened using inclusion and exclusion criteria. Once full texts were assessed for eligibility and 3 articles were included from manual search, 11 articles remained (Figure 1).

#### Data Extraction

Data were extracted from all included full‐text articles using an extraction form that included the following fields: study design, location, population, intervention, outcome, summary of findings, type of violence, and entry point of the study population into the health sector.

### Methodological Quality of Included Studies

Quantitative and qualitative studies were assessed separately for methodological quality. A Critical Appraisal Skills Programme (CASP) quality assessment tool (qualitative checklist) was used to appraise the methodological quality of qualitative studies and qualitative aspects of mixed‐method studies. The qualitative studies were appraised for quality using a score between 0 and 2 for 10 questions, for a maximum score of 20. Qualitative studies most frequently lost points for not discussing the role of the researcher, not fully describing data analysis methods, or an unclear statement of findings. An adapted CASP tool was used to appraise the methodological quality of quantitative studies and quantitative aspects of mixed‐methods studies (Oram et al. 2012). The quantitative studies were appraised for quality using a score between 0 and 2 for 15 questions, for a maximum score of 30. Quantitative studies most frequently lost points for not discussing ethical issues, confounders, or nonparticipation. The quality assessment tools were used to rate and describe the quality of the studies. However, no studies were excluded based on methodological reasons, and we used the scores to primarily assess the strengths and weaknesses of each study. Some studies had poor quality but were included (Guedes et al. 2002; Jacobs and Jewkes 2002) because of their strength in discussing the integration of health sector responses to gender‐based violence (GBV). This allowed us to discuss some types of interventions for which there was a lack of good quality evidence, but which nevertheless seemed an interesting intervention to test further. This also enabled a review of a much larger range of interventions (Ogilvie et al. 2005; Thomas and Harden 2008). Inclusion of all studies regardless of quality has been done by other literature reviews using thematic synthesis (Thomas and Harden 2008). Therefore, the quality assessment tools were not used for inclusion or exclusion from this review. However, quality assessment tools were still applied and used to enable informed judgments about the utility of studies and to examine the relative contributions of studies to the recommendations (Ogilvie et al. 2005; Thomas and Harden 2008).

#### Data Analysis

We analyzed qualitative and quantitative data separately, and the findings were combined into a final synthesis. The three quantitative studies reviewed had different aims and outcomes, therefore a descriptive synthesis was performed in lieu of a meta‐analysis. Thematic synthesis with a conceptual framework was used to identify the barriers and facilitators of integration of these interventions into the health sector. A thematic synthesis was used to go beyond the studies to generate new explanations and to examine factors influencing intervention implementation (Thomas and Harden 2008). The data analysis methods used for this review did not focus on the measure of effectiveness of these health sector responses to IPV, but enabled exploration of the contextual factors and processes of integrated IPV health sector responses.

#### Conceptual Framework

Our thematic analysis was informed by an adapted “health systems wheel” framework (part of health service delivery) (Colombini et al. 2012; Garcia‐Moreno et al. 2015) to extract data and analyze themes related to the integration of health sector responses to IPV in LMICs. We consolidated six health systems elements for data extraction and analysis: (1) leadership and governance (laws and policies on violence); (2) clear protocols and guidelines on violence; (3) health workforce development (e.g., training, supervision, and monitoring); (4) health infrastructure (e.g., privacy and confidentiality); (5) coordination (referral networks, links with non‐health‐sector agencies); (6) financing and budget allocation. Not covered as part of the review was Information Systems, because no studies covered this systems element. Comprehensive integrated responses were defined as having all or most elements in the conceptual framework addressed, while less comprehensive integrated responses were defined as having only a few elements. Selected interventions were ranked by level of comprehensiveness based on the elements of the framework.

## RESULTS

Focusing on the process of implementation and analyzing studies with a framework that ranked interventions by entry points and level of comprehensiveness enabled facilitators and barriers to integration to emerge. First, the study characteristics are presented. Then, the nature of integration by entry point is examined. Finally, findings regarding barriers and facilitators to integration are explored and organized by themes from the conceptual framework.

### Study Characteristics

Of the 11 studies retrieved, five used qualitative methodology (Guedes et al. 2002; Jacobs and Jewkes 2002; Colombini et al. 2012; Joyner and Mash 2012a; Rees, Zweigenthal, and Joyner 2014), three used quantitative methodology (Tiwari et al. 2005; Cripe et al. 2010; Matseke and Peltzer 2013), and three used mixed‐methods methodologies (Naved et al. 2009; Joyner and Mash 2012b; Turan et al. 2013). Six studies were conducted in Africa: five in South Africa (Jacobs and Jewkes 2002; Joyner and Mash 2012a and 2012b; Matseke and Peltzer 2013; Rees, Zweigenthal, and Joyner 2014) and one in Kenya (Turan et al. 2013). Three studies were conducted in Asia: in Bangladesh (Naved et al. 2009), Hong Kong (Tiwari et al. 2005), and Malaysia (Colombini et al. 2012). Two studies were conducted in the Caribbean and Latin America (Brazil, Dominican Republic, Peru, and Venezuela) (Guedes et al. 2002; Cripe et al. 2010). Seven studies discussed a health sector response to IPV (Tiwari et al. 2005; Cripe et al. 2010; Joyner and Mash 2012a and 2012b; Matseke and Peltzer 2013; Turan et al. 2013; Rees, Zweigenthal, and Joyner 2014) and four studies discussed joint responses to both sexual violence by nonpartners and IPV (Guedes et al. 2002; Jacobs and Jewkes 2002; Naved et al. 2009; Colombini et al. 2012). Table 2 describes study characteristics.

**Table 2:** Quantitative, qualitative, and mixed‐methods study characteristics

Study (Author, year)	Location	Study design	Study population	Intervention	Summary of relevant results	Type of violence	Entry points
**Quantitative study characteristics (3)**							
Cripe et al. 2010	Peru	Randomized two‐arm trial	220 abused pregnant women (aged 18–45) at ANC	IPV screening, referral card, and social worker case management (supportive counseling education and safety advice). Delivered by 4 hospital‐based social workers trained prior to the intervention.	Women in the empowerment training group tended to adopt more safety behaviors when compared with women in the standard care group. No statistically significant differences between control and intervention groups in health‐related quality of life, adoption of safety behaviors, and use of community resources.	IPV	ANC (hospital)
Matseke and Peltzer 2013	South Africa	Pre/post‐intervention design	Pregnant women presenting at PHC clinics for HIV post‐test counseling	18 community workers were trained in screening for IPV, and provided care, guidance, and referral to services.	7.2% of women screened positive for IPV. A statistically significant decrease in danger assessment score was found post‐intervention: the mean danger assessment score was 6.0 before intervention and fell to 2.8 post‐intervention (3 months).	IPV	ANC/PMTCT (PHC)
Tiwari et al. 2005	Hong Kong	Randomized controlled trial	Pregnant women experiencing IPV seeking ANC	Empowerment intervention including advice and empathetic understanding (one‐to‐one 30‐minute session and a brochure reinforcing information discussed, also on referrals).	Intervention group had higher physical functioning, less psychological abuse, lower postnatal depression scores than control group. No differences in severe physical violence and sexual abuse between intervention and control groups.	IPV	ANC
**Qualitative study characteristics (5)**							
Colombini et al. 2012	Malaysia	Qualitative study	Health workers and policy‐makers	Comprehensive medical care and counseling service. Internal referral to specialized services and external referral to police and social services.	Comprehensive care varied due to institutional constraints, management support, lack of human resources, training, protocols, and referral options.	IPV & SV	ED
Guedes et al. 2002	Dominican Republic, Peru, and Venezuela	Evaluation study—qualitative. Focus group discussions and in‐depth interviews.	Clients, service providers, and managers	Training health providers to detect, treat, and refer GBV survivors, improving institutional response to women who experience violence, collaboration with other organizations, raising community awareness about GBV.	Improved recognition of violence by health workers; improved privacy, confidentiality, and referrals. Some health workers were still disrespectful or judgmental to women.	IPV & SV	PHC/SRH
Jacobs and Jewkes 2002	South Africa	Qualitative. Focus groups.	Primary health care staff	Training health workers on identification and management of women experiencing GBV, referrals, and support.	Participants reported the training as motivating, informative, and empowering. Human resource shortages were a challenge.	IPV & SV	PHC
Joyner and Mash 2012a	South Africa	Qualitative. Focus groups and key informant interviews.	Primary health care providers, managers, academics, NGO leaders	Development and implementation of a protocol for screening and management of women experiencing IPV.	A cooperative inquiry process group produced a model of care for women experiencing IPV: case finding, clinical, psychological, social, and legal care.	IPV	PHC
Rees, Zweigenthal, and Joyner 2014	South Africa	Qualitative evaluation. Semi‐structured interviews and focus group discussions.	Health workers, women, and health managers	Comprehensive service model for IPV (identification and treatment, referral to IPV dedicated service for psychosocial‐legal care). Implemented in rural district.	Health workers’ barriers included: IPV normalization in the study community, poor understanding of the complexities of living with IPV, frustration in managing IPV cases. Health system constraints affected continuity of care, privacy, and integration of the intervention into routine functioning, and the intersectoral collaboration process was hindered by the formation of alliances.	IPV	PHC
**Mixed‐methods study characteristics (3)**							
Joyner and Mash 2012b	South Africa	Mixed methods. Cross‐sectional study, key informant interviews and focus group discussions.	Women experiencing IPV seeking primary care, health workers, and managers.	Health workers were trained on screening for IPV and referred cases to research nurse on site (“IPV champion”).	Health workers were reluctant to screen for IPV, hesitant to deal with complex and time‐consuming issues. However, committed providers continued screening.	IPV	PHC
Naved et al. 2009	Bangladesh	Mixed methods. Cross‐sectional study, in‐depth interviews.	Pregnant women experiencing IPV or SV, interviewed postnatally.	Training paramedics in mental health counseling to help abused women manage stress, improve coping, and enhance well‐being.	92% of women rated efforts in maintaining privacy good or very good; 99% said paramedics were not judgmental and 87% said the session improved self‐confidence.	IPV & SV	ANC/PHC (NGO based)
Turan et al. 2013	Kenya	Mixed methods. Cross‐sectional study, focus group, and in‐depth interviews.	Pregnant women seeking ANC, clinic staff, and community volunteers.	IPV risk assessment, medical care, and supported referrals for pregnant women experiencing violence.	53% of women experiencing IPV accepted referrals. Health workers saw benefits of screening, felt empowered and helpful, requested further training. Community collaboration helped with referrals and finding local solutions for clients; delays occurred with legal and justice systems. Community awareness on GBV increased.	IPV	ANC (PHC)

### Entry Points for Integrating IPV Responses in the Health Sector

There were various entry points at which IPV services were integrated into the health sector. Five studies examined antenatal care (ANC) (Tiwari et al. 2005; Naved et al. 2009; Cripe et al. 2010; Matseke and Peltzer 2013; Turan et al. 2013); one study examined care in emergency departments (Colombini et al. 2012); five studies examined primary health care (PHC) (Guedes et al. 2002; Jacobs and Jewkes 2002; Joyner and Mash 2012a and 2012b; Rees, Zweigenthal, and Joyner 2014), of which three implemented the same comprehensive model of IPV care. Typically, entry points for violence tend to be concentrated in ANC because of the screening model used in these services, also widely implemented in high‐income countries. In our analysis, ANC services are singled out as a PHC entry point because (1) most women in LMICs attend ANC and most LMIC women will become pregnant at least once, so it reaches most women at some point in their lives (which other PHC services do not), and (2) violence can often start or escalate in pregnancy and has consequences for the unborn child as well (Donovan et al. 2016).

Hospital emergency departments are also common entry points for joint IPV and sexual violence services, especially when severe physical injuries and rape are presenting conditions. Moreover, the model of the One‐Stop Crisis Centre was originally implemented in emergency departments.

More recently, IPV services have also been incorporated elsewhere in PHC, where the continuity of PHC offers general practitioners a chance to identify abused women presenting with chronic symptoms that could mask experiences of abuse. In our study, family planning (FP) clinics are included as PHC settings. However, FP clinics are highly specialized and are often provided by NGOs rather than the public sector.

#### Antenatal Care

Antenatal care was explored as an entry point for IPV response in five studies. Two studies examined ANC in hospitals (Tiwari et al. 2005; Cripe et al. 2010) and three studies in primary care (Naved et al. 2009; Matseke and Peltzer 2013; Turan et al. 2013). The main focus of the interventions was on screening for IPV and psychosocial support. Two ANC‐based interventions (Cripe et al. 2010; Turan et al. 2013) were more comprehensive (e.g., intersectoral response, training). Moreover, the Kenyan study had a strong link with community‐level response (through community volunteers) and prevention activities (through community awareness). The community awareness involved meeting with local leaders to discuss IPV and promoting anti‐violence messages in the community (Turan et al. 2013), which resulted in increased community awareness of gender‐based violence and identification of local solutions for support in rural areas where such services are scarce.

#### Emergency Department

Emergency departments were used as entry points in one study that assessed the comprehensive model of One‐Stop Crisis Centres (Colombini et al. 2012). Its on‐site and 24‐hour violence responses involved dedicated staff, a private location, available procedures, coordination with the intersectoral response, referrals, and health worker training.

#### Primary Health Care

Five studies discussed primary health care (general outpatient services) as an entry point for response to IPV (Guedes et al. 2002; Jacobs and Jewkes 2002; Joyner and Mash 2012a and 2012b; Rees, Zweigenthal and Joyner 2014). These interventions focused on in‐service training for health workers and identification of women experiencing IPV. Four studies (two part of the same intervention and one piloting the same model) were comprehensive and focused on IPV screening, in‐service training (Joyner and Mash 2012a), the implementation of clinical guidelines, documentation, referral to supports and safety planning (Joyner and Mash 2012b; Rees, Zweigenthal, and Joyner 2014), improving institutional response, coordination with other organizations, and raising awareness about violence against women (Guedes et al. 2002). One less comprehensive study focused primarily on screening and training, although added engagement with management and NGOs (Jacobs and Jewkes 2002). It is also worth noting that one study that took place in FP clinics was implemented by NGOs rather than the public sector (Guedes et al. 2002).

### Barriers and Facilitators to Integration

We used a health systems elements conceptual framework to examine the degree of integration in the studies. We wanted to explore how comprehensive and integrated the health sector responses were based on a scoring of how far the response covered each of the six health systems elements of our framework. When four or more elements were implemented in the study, the program was considered comprehensive and integrated. Three or less elements meant the program was considered less comprehensive and integrated. Results are shown in Table 3. One pair of studies describing the same individual interventions and one piloting the same intervention model were combined (Joyner and Mash 2012a and 2012b; Rees, Zweigenthal, and Joyner 2014).

**Table 3:** Integration scores for the health systems responses in each study

	Colombini et al. 2012	Cripe et al. 2010	Guedes et al. 2002	Jacobs and Jewkes 2002	Joyner and Mash 2012a/b; Rees, Zweigenthal, and Joyner 2014	Matseke and Peltzer 2013	Naved et al. 2009	Tiwari et al. 2005	Turan et al. 2013
**Entry point**	ED	ANC	PHC	PHC	PHC	ANC	ANC	ANC	ANC
**Type of violence**	IPV/SV	IPV	IPV/SV	IPV/SV	IPV	IPV	IPV/SV	IPV	IPV
Leadership and guidance at management level	√		√	√	√				
Policies and protocols on violence at service delivery	√	√	√		√	√			√
Health infrastructure (setting that enables privacy, confidentiality, and safety)	√	√	√		√		√		√
Health workforce development (e.g., training staff and having designated staff)	√	√	√	√	√	√	√	√	√
Coordination	√	√	√	√	√	√			√
Financing (budget allocation)									
Integration Score	**C**	**C**	**C**	**LC**	**C**	**LC**	**LC**	**LC**	**C**

ANC = Antenatal Care. C = Comprehensive. ED = Emergency Department. IPV = Intimate Partner Violence. LC = Less Comprehensive. PHC= Primary Health Care. SV = Sexual Violence.

#### Comprehensive and Integrated Health Sector Responses to IPV

Seven studies showed comprehensive integrated responses to IPV, having four or more crucial systems elements in place. One was based in emergency departments, four in PHC, and two in ANC. Findings show that the common facilitators to a more comprehensive response are: clear and well‐implemented guidelines and protocols; leadership at a management level, manifest in commitment and support; sensitized and trained health workers receiving ongoing training and a supportive environment; and coordination across the health sector and beyond with clear referral structures and high‐quality referral services. Financing and budget allocation (the sixth health systems element) did not feature in our study framework. This may be because most of these studies were pilot studies and did not take into account budget implications for integration.

#### Clear Policies and Protocols on Violence at Service Delivery

Six studies reported on availability of specific policies and protocols for IPV. A study on family planning services in Latin America demonstrated how clear policies on confidentiality and privacy contributed to a culture of respect within PHC clinics (Guedes et al. 2002).

Conversely, the lack of guidelines or poor implementation of guidelines arose as a barrier to the integration of health sector responses to violence. In Malaysia, guidelines were developed but were not fully implemented or were not specific enough, leading to health workers reporting uncertainty about care for women who had experienced IPV in an otherwise well‐integrated emergency department setting (Colombini et al. 2012).

#### Leadership and Guidance

An important aspect evident in four studies with comprehensive responses to violence (three in PHC and one in an emergency department) was the leadership of, and engagement with, management in the health system to develop and implement interventions. A comprehensive PHC model in a rural district in South Africa established an implementation team consisting of managers from the Department of Health, a supervisor from the Department of Social Development, and a representative of the Police Service (Rees, Zweigenthal, and Joyner 2014). Providing training to both managers and health workers in primary care in South Africa led to increased recognition of IPV as a health problem and increased support to health workers by management (Joyner and Mash 2012a). Another PHC‐based intervention in Latin America found that changing the hiring policy to ensure that management reflected gender‐equality values, along with training health workers, created a supportive environment (Guedes et al. 2002).

#### Human Workforce Development

Many enabling and constraining factors to a comprehensive health sector response to IPV related to health workers.

The training of health workers was found to be an enabling factor in a comprehensive health sector response. All comprehensive interventions trained health workers through in‐service training. One study trained health workers on the use of clinical guidelines, management of women who experienced rape, forensic evidence collection, and available referral options (Colombini et al. 2012). One study provided further training to motivated health workers to build their IPV expertise and help them become “IPV champions.” A study in South Africa found that care for women who experienced IPV was very time‐consuming, so a member of the multidisciplinary team was identified as being an IPV champion. This role required empathy, good communication, and respect for confidentiality. All health workers received training, screened women for IPV, and provided clinical care. The “IPV champion” then provided further care involving risk assessment, safety planning, and referrals to psychosocial, legal, and police services (Joyner and Mash 2012b).

It became apparent from the findings that health workers’ attitudes hindered comprehensive responses to IPV. Negative attitudes among health workers were cited as a barrier to the provision of comprehensive health care for women experiencing violence (Guedes et al. 2002; Jacobs and Jewkes 2002; Rees, Zweigenthal, and Joyner 2014). Negative attitudes among health workers made discussing IPV uncomfortable, as did being judgmental or blaming women who experienced IPV, and there was often poor recognition of IPV as a health problem. These negative attitudes decreased the uptake of screening, clinical inquiry, or protocols for the provision of supportive care and referral to further support by health workers. Another barrier to a comprehensive health sector response to IPV was lack of ongoing training, and lack of support following initial training. This was either self‐reported by staff (Colombini et al. 2012), or the lack of proficiency or competence following initial training indicated the need for further training (Joyner and Mash 2012b).

#### Coordination

Most studies referred women to on‐site or off‐site specialized health services, or legal, social, or justice services. All of the more comprehensive interventions developed formal referral structures. Emergency department–based services provided referral, based on a protocol, to on‐site counselors (Colombini et al. 2012). Referral to off‐site support services is a key element of an intersectoral response requiring strong coordination. Five of the interventions (one emergency department, two ANC, and two PHC) demonstrated how the linkages between referral structures and a well‐coordinated intersectoral response facilitate comprehensive responsiveness to IPV (Guedes et al. 2002; Cripe et al. 2010; Colombini et al. 2012; Turan et al. 2013; Rees, Zweigenthal, and Joyner 2014).

Coordination within an intersectoral response and within the health sector emerged as another component that facilitated comprehensiveness. Well‐integrated emergency department responses coordinated within the health sector to facilitate internal referrals to specialist services (Colombini et al. 2012). Comprehensive health sector responses collaborated across multiple sectors through regular multisectoral meetings in the program development, implementation, or throughout provision (Guedes et al. 2002; Colombini et al. 2012). Multisectoral collaboration facilitated integration by improving support for the intervention, and police reporting (Colombini et al. 2012). For example in Malaysia, the regular meetings of a coordinating committee facilitated successful external referral systems (Colombini et al. 2012).

A lack of high‐quality referral options, particularly in low‐resource or rural areas, emerged as a barrier to integration (Rees, Zweigenthal, and Joyner 2014). Some innovative solutions for improving referral options arose. One study suggested that community‐based support groups might be a beneficial referral option in rural settings (Joyner and Mash 2012b). Two studies found that having a community volunteer accompany women to external support services and provide emotional support improved women's ability to access services further away or perceived as judgmental, ineffective, or corrupt (Guedes et al. 2002; Turan et al. 2013). Another study trained community leaders and referred women or their partners to these community leaders for support or counseling (Turan et al. 2013).

Coordination between different sectors requires sustainable funding and clarity in the roles and responsibilities of each sector. Conversely, the lack of clear roles in each sector emerged from these findings as a barrier to integration. This can be illustrated in Malaysia, where counseling and support services were provided by NGOs and clinical services by emergency department health workers. Initially, a lack of clarity regarding the role of NGOs and emergency department staff created a barrier to comprehensive care. However, this barrier was reduced when a joint protocol detailing each actor's role was developed (Colombini et al. 2012). In South Africa, different management support levels and rigid management hierarchies, decentralization of control and availability of resources, and lack of clarity regarding intersectoral partners’ roles were also seen to be challenges (Rees, Zweigenthal, and Joyner 2014). Although formal structures and roles of intersectoral partners were found to be important, the effects of informal relationships and communication, and shared ownership across individual actors were more significant. In addition, the tight parameters within which managers and service providers are required to operate contrasted with the need for fluid intersectoral collaboration (Rees, Zweigenthal, and Joyner 2014).

#### Financing and Budget Allocation

Only two studies mentioned paucity of financial (and human) resources as significant issues affecting the implementation of services. One South African study explicitly reported scarce resources for the pilot study, which affected the availability of the intervention. In fact, the intervention could only be provided once a month at each PHC facility, because one service provider had only ten days a month to dedicate to the intervention, which was carried out over a large geographical area (Rees, Zweigenthal, and Joyner 2014). In Malaysia, the limited budget committed to One Stop Crisis Centres affected the implementation of IPV services, especially in subdistrict facilities (Colombini et al. 2012).

A summary of barriers and facilitators is detailed in Table 4.

**Table 4:** Comprehensive and integrated services and systems response: Barriers and facilitators

Study of comprehensive services	Barriers to integration	Facilitators to integration
Colombini et al. 2012	Lack of support at management level. Clinical guidelines were developed, but not always used. Referral options were limited, coordination was dysfunctional, little clarity on roles of different actors. Training availability was limited; too focused on sexual violence, forensic evidence, and medical treatment; lacked focus on intimate partner violence. Lack of health worker supervision.	Comprehensive services such as counseling, medical care, support services, police and collection of forensic evidence, legal aid, and temporary shelter were provided. Internal referral systems and interagency network‐facilitated referrals and collaboration. In some instances, “IPV champions” (e.g., head of emergency department) supported the response.
Cripe et al. 2010	Ineffective justice and police systems leading to low uptake of community resource referral and abused women seeking help from informal sources. IPV laws and policies have not been fully implemented or enforced.	Not documented
Guedes et al. 2002	Despite training, certain health care professionals were disrespectful of women because of professional, class, ethnic, or gender hierarchies. Sustaining costs for lawyers in‐house and for staff time to accompany women to legal services.	Clients’ privacy and confidentiality was respected through improved physical infrastructure, adjusting client flow, and revising policies to protect client records. Training that addressed participants’ own beliefs and concerns regarding IPV (taking human rights perspective). Management support on IPV (e.g. policies on recruitment changed—new staff asked about IPV). Referrals: strengthened referrals to legal services, and some clinics hired a lawyer in‐house. Women found it helpful when clinic staff accompanied them to legal services. External support from other GBV networks: joined local‐ and national‐level networks to advocate for legal and judicial reforms on IPV.
Joyner and Mash 2012a	Nurses felt overwhelmed and unsupported and needed to protect themselves from further demands from clients and managers.	Providers were equipped with a laminated list of possible screening questions. Easy access to on‐site support via “IPV champions.” Training of all staff (including managers) led to increased management support for implementing the intervention. Referrals from study nurses increased women's chances of accessing legal services.
Joyner and Mash 2012b	Reluctance to screen because of: possible personal experiences with IPV; could take extra time; fear of invading clients’ privacy and being targeted by partners; perception of IPV as a social not biomedical problem; busy and heavy workload; lack of knowledge and skills to manage mental health issues.	Demonstrated commitment of some health providers.
Rees, Zweigenthal, and Joyner 2014	Limited availability: Intervention provided only once a month due to a lack of resources, with one service provider having ten days a month to dedicate to the intervention. Timing of the intervention was also problematic for the same reason, leading to long wait (up to a month) from time of referral. Poor intersectoral collaboration due to lack of resources and support. Lack of management support; organizational limitations. Lack of structured follow‐up system. Negative attitudes of health workers toward IPV, and limited mental health knowledge and skills among social workers.	Referrals to mental health services were high (although referral pathways were not always effective). Existence of formal structures of intersectoral collaboration (e.g., intersectoral committee), informal relationships and communication, as well as shared ownership, were found significant.
Turan et al. 2013	Some male health workers and community volunteers were criticized by other community members for involvement in GBV services. Most participants stressed the need for repeated refresher trainings and sensitization for service providers and local partners (including local administration and police) as well as additional counseling skills for community volunteers and health workers. Criminal and legal proceedings could not be completed in this area, but in the next town, causing delays in pressing charges. Screening declined over time and clinicians used “case finding” (assessing some clients and not others) instead. Limited funds were available to: support transport costs for clients and community volunteers to reach referral agencies in the nearest town; cover cell phone costs for health workers and volunteers so they could communicate with each other, and for referral agencies and biweekly meetings of volunteers.	Community‐level collaboration to increase awareness of services for and harms of GBV. Community involvement increased potential support available in a low‐resource and rural setting. All clinic staff were trained, including administrators, increasing the acceptance of the program and delivery of services. Nonclinicians were also involved in giving information and support. Supported referrals were available through community volunteers who escorted women to services and provided emotional support.

#### Less Comprehensive and Integrated Health Sector Responses to IPV

Four less‐integrated health sector responses were found: three based in ANC and one in PHC (see Table 5). Two intervention studies focused on identification of IPV and referral to psychosocial support during ANC. This psychosocial support involved an advocacy or empowerment intervention or brief mental health counseling (Tiwari et al. 2005; Matseke and Peltzer 2013). One intervention referred women from ANC to trained paramedics to offer mental health counseling for those who experienced violence (Naved et al. 2009). Another intervention developed and implemented a training model for addressing violence in the health sector (Jacobs and Jewkes 2002). These brief, small‐scale studies did not directly coordinate with the intersectoral response for their referrals, and did not mention management engagement or availability of guidelines, policies, and protocols to support the intervention, apart from two studies in South Africa that trained staff (health care workers and community workers) in a protocol for assessment and intervention for abuse during pregnancy (Jacobs and Jewkes 2002; Matseke and Peltzer 2013). Only one PHC study (*Vezimfilho* model) in South Africa involved management in the development of the intervention. In particular, training for PHC health workers was developed with health management and key NGO partners to ensure a supportive environment in which the training could be implemented and sustained (Jacobs and Jewkes 2002). However, trained staff were concerned that health managers would be a barrier to effective skills implementation.

**Table 5:** Less comprehensive and integrated services and systems response: Barriers and facilitators

Studies of less comprehensive services	Barriers to integration	Facilitators for integration
Jacobs and Jewkes 2002	Negative experiences were largely related to the broader health system, such as shortage of staff to resolve the problem. Fear that health managers would not be supportive of IPV skill implementation.	Government and Department of Health support. Training of health managers and key referral providers. Successful implementation required ongoing advocacy with health management to recognize IPV as a public health problem that needs health system support for PHC staff.
Matseke and Peltzer 2013	Not documented	Not documented
Naved et al. 2009	Long waiting time before seeing the paramedic. Some women reported that coming to counseling sessions at the clinic could escalate violence from their husbands.	Paramedics were empathetic, nonjudgmental, and treated women as equals. Privacy and confidentiality were maintained. Ongoing support and debriefings during the first six months of the intervention.
Tiwari et al. 2005	Not documented	Not documented

## DISCUSSION

This review synthesized studies of health sector responses to IPV in LMICs. Previous reviews were either in high‐income countries or were focused on particular aspects of effectiveness, entry points, or models of care, not processes or levels of integration (Taft et al. 2013; Bair‐Merritt et al. 2014; Garcia‐Moreno et al. 2015). Only one previous review on IPV screening studies assessed their comprehensiveness and suggested that successful interventions included institutional support, effective screening protocols, thorough initial and ongoing training, and immediate access/referrals to on‐site and/or off‐site support services (O'Campo et al. 2011). Focusing on the process of implementation and analyzing studies with a framework that ranked interventions by entry points and level of integration and comprehensiveness allowed a better understanding of facilitators and barriers to comprehensive IPV responses. Study entry points were emergency departments, PHC, and ANC. The most comprehensive studies in responsiveness to IPV were based in PHC and ANC. Only one study in an emergency department was fully integrated. Findings emerging from this review suggest the following facilitators to comprehensive care: availability of clear guidelines, policies, or protocols; management support; intersectoral coordination with clear, accessible on‐site and off‐site referral options; adequate and trained staff with accepting and empathetic attitudes toward women who have experienced IPV; initial and ongoing training for health workers; and a supportive and supervised environment in which to enact new IPV protocols.

Irrespective of the service entry point, what emerged as crucial is a connected systems‐level response, with each element implemented in a coordinated manner. A key characteristic of the most comprehensive responses was the connection or “linkages” (whether made consciously or not) between different individual factors, rather than a single element. For example, clinical guidelines, protocols, or policies were more effectively implemented when there was also managerial support and leadership, when an intersectoral response was made or coordination within the health sector ensured. Individually, these elements may not result in a comprehensive health sector response, but together management support, multisectoral partnership involvement, and clinical guideline development and implementation can facilitate a comprehensive response. PHC and ANC units emerged as the primary entry points delivering the most comprehensive integrated care (Table 3). In most studies across these entry points, multiple health systems elements that emerged as important were addressed. Moreover, in the highest‐scoring studies, linkages between elements were explicit. The links between each facilitating element thus seem pivotal. For example, management involvement and support to staff throughout an intervention facilitates the clear implementation of guidelines within the health settings. This was the case in tertiary hospitals in Malaysia, where trained nurses and doctors had the support of emergency department heads (Colombini et al. 2011; Colombini et al. 2012). In South Africa, comprehensive training of all staff (including managers) at PHC clinics was a strategy that led to increased management support (Joyner and Mash 2012a and 2012b). Coordination within the intersectoral response facilitates the development of a referral structure and improved access to support services for women experiencing IPV. Clear partner roles, continuous consultations, equal representation, and sense of ownership are significant to improve and sustain collaborations (Rees, Zweigenthal, and Joyner 2014).

Reflecting on the health systems elements captured in our conceptual framework, four emerged as important facilitators of integration for comprehensive health sector responses to IPV in the studies in this review: clear policies; leadership; coordinated referrals and inter‐sectoral collaboration; and training and support for health workers. Combined, these elements should facilitate a comprehensive response.

Policies emerged as important only as a small “p”—clear service‐delivery guidelines, protocols, and clinic‐level policies that guided implementation were shown, across entry points, to improve quality of care, treatment, and legal documentation of violence cases. Incorporating screening protocols with clear guidelines on how providers could address IPV was also influential in high‐income country studies to reduce providers’ fear of asking women about violence, and to improve providers’ self‐efficacy (O'Campo et al. 2011). No studies commented on the available legal framework (big “P” policies) regarding violence against women, possibly because the policies pre‐existed the interventions. However, its importance in legitimizing the intervention and the work of motivated providers is crucial (Colombini et al. 2011; Goicolea et al. 2015).

Leadership and governance was revealed to be important specifically in relation to the management tier. The engagement of management and leadership, as well as the initiative shown by managers, was found to be important in both emergency department and PHC settings. It is possible that engaging management and administration staff at the outset of an intervention or proposed change motivates staff to lead and take initiative to be creative, support a comprehensive approach, and improve skill implementation. The “*Dilaasa* model,” a hospital‐based crisis center piloted in India, also shows how the support of key hospital functionaries, such as the medical superintendent, was crucial to the sustainability of the model, for example by assenting and subsequently leading the center as part of the hospital structure, by deploying staff for training and subsequent responsibilities, and by ensuring that other facilities and resources were made available (Ravindran and Undurti 2010). However, clearly not all managers are leaders and more research is needed to better understand what leadership qualities are needed in management cadres and how these can be nurtured.

A number of human resource/training factors emerged as important. Ensuring trust and confidentiality are crucial elements for implementing a successful health response to IPV, especially in PHC and ANC settings, where providers are able to build long‐term relationships with their clients. Ongoing and continuous training, including in areas that typically go beyond nurses’ scope and clinical skills, such as forensic exams, was found to be important for IPV response because it can more effectively connect to other needed sectors (e.g., police, justice, counseling). Aside from the clinical aspects of the IPV protocol, staff training should address attitudes among staff that are negative (such as blaming IPV survivors) and should increase the staff's ability to help abused women. Additionally, the presence of a trained and motivated IPV champion to motivate and support other health workers and provide further specialist care was found to be very beneficial in both PHC and emergency department settings in Malaysia (Colombini et al. 2012), South Africa (Joyner and Mash 2012b), and India (Krishnan 2014). The presence of IPV advocates on‐site linking the health and nonhealth sectors also proved beneficial in increasing providers’ identification and referrals in other studies (Feder et al. 2011). Involving an NGO to co‐lead the health response to address violence also proved efficient in the Dilaasa crisis intervention center in India, especially when training existing hospital staff to respond to violence against women and integrating a violence service response into their roles and responsibilities (Bhate‐Deosthali, Ravindran, and Vindhya 2012).

However, training alone has proven unable to change providers’ practices in the long run (Garcia‐Moreno et al. 2015). The Vezimfilho study showed that to be sustainable, health interventions should build staff capacity both on interpersonal and professional levels, and also be supported by health managers (Jacobs and Jewkes 2002). Furthermore, clear, effective, and coordinated referral pathways both within and beyond the health sector emerged as important across all entry points. Intersectoral collaboration is especially important for a comprehensive response to IPV, because the nature of the collaboration requires attention by sectors beyond the health sector, including police and justice, social welfare, and community services (Colombini, Mayhew, and Watts 2008; García‐Moreno et al. 2015). Referral structures that link women to support services increase the likelihood of identification by the health sector (McCaw et al. 2001), even among less motivated staff (Goicolea et al. 2015). In addition, linking health services with community services and volunteers can help improve community awareness of violence and offer additional support services in rural areas where these are scarce (Turan et al. 2013). Supportive environments at the community level are important. At the Soukhya Project in India, community health workers and project staff created community awareness on violence and referral services in tandem with offering GBV services at PHC settings (Krishnan 2014). Referral networks and coordinated responses have to be sustained, however, and available support services should be continuously mapped and reassessed (through client feedback) to ensure quality of services.

Health infrastructure, primarily the ensuring of women's privacy and confidentiality, only emerged as a facilitator in one study (Guedes et al. 2002). Health information systems were not documented. This could have been because such systems were simply not considered in the studies, except in relation to referrals, although this seems unexpected given the need for data sharing between sectors that is required in particular for justice considerations and to monitor progress and quality of care (García‐Moreno et al. 2015). One explanation may be that the majority of the studies in this review were pilot studies and health information and monitoring considerations are typically addressed only at scale‐up.

Similarly, most studies did not mention financing and budget allocation. Again, an explanation may be that most were pilot or trial studies, so financing and budgeting was not an explicit issue. As pilot studies become scaled‐up, however, financial considerations will become very important. There is currently a dearth of research on scale‐up of IPV interventions. Moreover, specific budget allocation for IPV services helps create sustainable responses and represents a leadership commitment from senior health managers to address this important issue (García‐Moreno et al. 2015).

There is an urgent need for process evaluations to be published alongside future IPV trials because they can yield contextual information on how programs are implemented, which is also helpful in scaling up IPV interventions. Furthermore, process outcomes should be considered alongside impact outcomes on IPV reduction and women's mental health. The evidence to date suggests that ANC (in clinics) and PHC interventions were most comprehensive, though one emergency department–based response in hospitals also showed promising results. Other entry points such as HIV services (e.g., prevention of mother to child transmission, and HIV counseling and testing) have also been used to prevent and address partner violence (Christofides and Jewkes 2010; Anderson, Campbell, and Farley 2013). Whichever entry point is chosen, these review results show that addressing multiple and combined health systems elements by adopting a systems approach is necessary for achieving a comprehensive response.

### Limitations

One limitation of this review is the lack of methodologically strong studies; most studies were observational. Emerging from the quality assessment of the studies, short time‐frames were noted in post‐intervention assessments potentially exaggerating the effects of interventions. Evaluations were often self‐reported, creating a risk of information bias. In addition, there was a lack of studies presenting the processes around integration, as most studied the effectiveness of specific interventions. It is worth noting that if articles failed to present evidence for a certain health systems component, it does not necessarily mean that they failed to include the topic during their study. It could also be because of word count limitations imposed by journals, resulting in the deletion of details about the process of implementing these interventions. Future intervention studies should better document the processes used to implement specific programs, as these are crucial for scaling up.

In searching for peer‐reviewed articles and limiting the search to English, a publication bias was introduced. However, the focus on service entry points enabled a comparison of models of care and their responsiveness to IPV. This previously caused a gap in the literature, which this review aimed to fill.

## CONCLUSION

This article synthesizes studies of health sector responses to IPV in LMICs. A systems‐level response, addressing multiple systems elements at the same time, is critical to facilitate a comprehensive health sector response to IPV, with each element implemented together in a coordinated way. Facilitators and barriers to integration emerged and were discussed. Analyzing the different factors that enabled more comprehensive responses and constrained less comprehensive responses filled a gap in the literature. Focusing on the process of implementation and analyzing studies with a framework that ranked interventions by level of comprehensiveness allowed facilitators and barriers to emerge. Facilitating factors should be adapted and applied to each context as appropriate.

## Supporting information

Supporting Information

## Footnotes

1 Appendix is available at the supporting information tab at wileyonlinelibrary.com/journal/sfp.
