# Supplementary material for: Barriers and Facilitators to Integrating Health Service Responses to Intimate Partner Violence in Low‐ and Middle‐Income Countries: A Comparative Health Systems and Service Analysis
Source: Stud Fam Plann. 2017 Apr 19;48(2):179–200. doi: 10.1111/sifp.12021 (PMC5518204; doi:10.1111/sifp.12021)
Supplement: Supplementary file 1 — Supporting Information [file SIFP-48-179-s001.docx]

**Appendix: Full list of search terms and strategy for each database**

| **Search engine** | **# of 10 November 2015 results** |
| --- | --- |
| Global health | 571 |
| Medline | 579 |
| Embase | 712 |
| Cinahl | 346 |
| Web of Science | 2108 |
| Total | 4316 |

**Global health**

Search terms:

gender based violence or intimate partner violence or intimate partner abuse or spous$ violence or spous$ abuse or domestic violence or domestic abuse or family violence or sexual violence or batter$ wom#n or marital violence or dating violence or sexual assault or rape or violence against wom#n

Subject headings:

sexual abuse/

sexual assault/

“rape (trauma)”/

domestic violence/

spouse abuse/

1 or 2 or 3 or 4 or 5 or 6 or 7 or 8 or 9 or 10 or 11 or 12 or 13 or 14 or 15 or 16 or 17 or 18 or 19 or 20

health sector or health system? or health service? or health polic$ or health facilit$ or hospital? or clinic? Or primary health care or secondary health care or tertiary health care or health care or antenatal or reproductive health or sexual health

health services/

health care/

health policy/

health care workers/

1. 22 or 23 or 24 or 25 or 26 or 27 or 28 or 29 or 30 or 31 or 32 or 33 or 34 or 35 or 36 or 37 or 38 or 39

prevent? Or program? Or intervent$ or evaluat? or monitor? or implementat? or respon$ Or Review or screen? Or protocol? Or polic$ or guideline? Or train?

1. 41 or 42 or 43 or 44 or 45 or 46 or 47 or 48 or 49 or 50 or 51 or 52 or 53 or 54
2. developing countr$ or low income countr$ or middle income countr$
3. List of World Bank LMIC (Afghanistan or Benin or Burkina Faso or Burunid or Cambodia or Central African Republic or Chad or Comoros or Democratic Republic Congo or Eritrea or Ethiopia or Gambia or Guinea or Guinea Bisau or Hait or North Korea or Liberia or Madagascar or Malawi or Mali or Mozambique or Nepal or Niger or Rwanda or Sierra Leone or Somalia or South Sudan or Tanzania or Togo or Uganda or Zimbabwe or Armenia or Bangladesh or Bhutan or Bolivia or Cabo Verde or Cameroon or Congo or Cote d’Ivoire or Ivory Coast or Djibouti or Egypt or El Salvador or Indonesia or Kenya or Kiribati or Kosovo or Kyrgyz Republic or Lao or Laos or Lesotho or Mauritania or Micronesia or Moldova or Morocco or Samoa or Sao Tome Principe or Senegal or Solomon Islands or Sri Lanka or Sudan or Swaziland or Syria or Tajikistan or Georgia or Ghana or Guatemala or Guyana or Honduras or India or Myanmar or Nicaragua or Nigeria or Pakistan or Papua New Guinea or Philippines or Timor Leste or Ukraine or Uzbekistan or Vanuatu or Vietnam or West Bank or Gaza or Yemen or Zambia)
4. Africa south of sahara/
5. Central America/
6. South America/
7. 56 or 57 or 58 or 59 or 60 or 61 o 62
8. 21 and 40 and 55 and 63

Total: 571 on Nov 8, 2015

**MEDLINE**

(gender based violence) or (intimate partner violence) or (intimate partner abuse) or (spous$ violence) or (spous$ abuse) or (domestic violence) or (domestic abuse) or (family violence) or (sexual violence) or (batter$ wom#n) or (marital violence) or (dating violence) or (sexual assault) or (rape) or (violence against wom#n)

1. Domestic violence/
2. spouse abuse/
3. rape/
4. battered women/
5. 1 or 2 or 3 or 4 or 5 or 6 or 7 or 8 or 9 or 10 or 11 or 12 or 13 or 14 or 15 or 16 or 17 or 18 or 19

health sector or health system? Or health service? Or health polic$ or health facilit$ or hospital? Or clinic? or primary health care or secondary health care or tertiary health care or health care or antenatal or reproductive health or sexual health

Subject heading:

health facilities/

health personnel/

health services/

delivery of health care/

health policy/

comprehensive health care/

integrated health care/

1. 21 or 22 or 23 or 24 or 25 or 26 or 27 or 28 or 29 or 30 or 31 or 32 or 33 or 34 or 35 or 36 or 37 or 38 or 39 or 40 or 41

prevent? Or program? Or intervention or evaluation or monitoring or implementation or response? Or Review or screen? Or treat? Or protocol? Or polic$ or guideline? Or train?

1. 43 or 44 or 45 or 46 or 47 or 48 or 49 or 50 or 51 or 52 or 53 or 54 or 55 or 56
2. Medline’s expert search of LMIC
3. 20 and 42 and 57 and 58

Search on Nov 8, 2015: 579

**Embase**

gender based violence or intimate partner violence or intimate partner abuse or spous$ violence or spous$ abuse or domestic violence or domestic abuse or family violence or sexual violence or batter$ wom#n or marital violence or dating violence or sexual assault or rape or violence against wom#n

1. partner violence/
2. sexual abuse/
3. gender based violence/
4. rape/
5. domestic violence/
6. sexual assault/
7. sexual violence/
8. 1 or 2 or 3 or 4 or 5 or 6 or 7 or 8 or 9 or 10 or 11 or 12 or 13 or 14 or 15 or 16 or 17 or 18 or 19 or 20 or 21 or 22

health sector or health system? Or health service? Or health polic$ or health facilit$ or hospital? Or clinic? or primary health care or secondary health care or tertiary health care or health care or antenatal or reproductive health or sexual health

Subject heading:

health care facility/

health care delivery/

health care/

health care personnel/

health care policy/

1. 24 or 25 or 26 or 27 or 28 or 29 or 30 or 31 or 32 or 33 or 34 or 35 or 36 or 37 or 38 or 39 or 40 or 41 or 42

prevent? Or program? Or intervention or evaluat$ or monitor? or implement? or response? Or review or screen? Or treat? Or protocol? Or polic$ or guideline? Or train?

1. 44 or 45 or 46 or 47 or 48 or 49 or 50 or 51 or 52 or 53 or 54 or 55 or 56 or 57
2. developing countr$ or low income countr$ or middle income countr$
3. Africa south of sahara/
4. South America/
5. Central America/
6. List of World Bank LMIC
7. 59 or 60 or 61 or 62 or 63 or 64 or 65
8. 23 or 43 or 58 or 66

Total: 2119

Once limits English and after 2000 applied: 712

**CINAHL**

gender based violence or intimate partner violence or intimate partner abuse or spous* violence or spous* abuse or domestic violence or domestic abuse or family violence or sexual violence or batter* wom?n or marital violence or dating violence or sexual assault or rape or violence against wom?n

1. sexual abuse/
2. intimate partner violence/
3. domestic violence/
4. dating violence/
5. battered women/
6. 1 or 2 or 3 or 4 or 5 or 6 or 7 or 8 or 9 or 10 or 11 or 12 or 13 or 14 or 15 or 16 or 17 or 18 or 19 or 20
7. health sector or health system* or health service* or health polic* or health facility* or hospital* or clinic* or primary health care or secondary health care or tertiary health care or health care or antenatal or reproductive health or sexual health

health facilities/

health care delivery/

health care delivery, integrated/

health policy/

health personnel/

health services/

1. 22 or 23 or 24 or 25 or 26 or 27 or 28 or 29 or 30 or 31 or 32 or 33 or 34 or 35 or 36 or 37 or 38 or 39 or 40 or 41

Prevent* or program* or intervention or evaluat* or monitor* or implement* or response* or review or screen* or protocol* or polic* or guideline* or train*

1. 43 or 44 or 45 or 46 or 47 or 48 or 49 or 50 or 51 or 52 or 53 or 54 or 55

low income countr* or middle income countr* or developing countr*

1. List of Work Bank LMIC
2. “Developing countries”/
3. “Africa South of the Sahara”/
4. “South America”/
5. “Central America”/
6. 57 or 58 or 59 or 60 or 61 or 62 or 63 or 64
7. 21 and 42 and 56 and 65

Total: 1,472

Once limits English and after 2000 applied: 346

**Web of Science**

Gender based violence or intimate partner violence or intimate partner abuse or spous* violence or spous* abuse or domestic violence or domestic abuse or family violence or sexual violence or batter* wom?n or marital violence or dating violence or sexual assault or rape or violence against wom?n

1. 1 or 2 or 3 or 4 or 5 or 6 or 7 or 8 or 9 or 10 or 11 or 12 or 13 or 14 or 15

Health sector or health system* or health service* or health polic* or health facilit* or hospital* or clinic* or primary health care or secondary health care or tertiary health care or health care or antenatal or reproductive health or sexual health

1. 17 or 18 or 19 or 20 or 21 or 22 or 23 or 24 or 25 or 26 or 27 or 28 or 29 or 30

Prevent* or program* or intervention or evaluat* or monitor* or implement* or response* or review or screen* or protocol* or polic* or guideline* or train*

1. 32 or 33 or 34 or 35 or 36 or 37 or 38 or 39 or 40 or 41 or42 or 43 or 44
2. low income countr*
3. middle income countr*
4. developing countr*
5. 46 or 47 or 48
6. 16 and 32 and 45 and 49

Once limits English and after 2000 applied: 2108
